# Induction of Protease Release of the Resistant Diatom *Chaetoceros didymus* in Response to Lytic Enzymes from an Algicidal Bacterium

**DOI:** 10.1371/journal.pone.0057577

**Published:** 2013-03-04

**Authors:** Carsten Paul, Georg Pohnert

**Affiliations:** Institute for Inorganic and Analytical Chemistry, Department for Bioorganic Analytics, Friedrich Schiller University, Jena, Germany; University of South Florida College of Medicine, United States of America

## Abstract

Marine lytic bacteria can have a substantial effect on phytoplankton and are even capable to terminate blooms of microalgae. The bacterium *Kordia algicida* was reported to lyse cells of the diatom *Skeletonema costatum* and several other diatoms by a quorum sensing controlled excretion of proteases. However the diatom *Chaetoceros didymus* is fully resistant against the bacterial enzymes. We show that the growth curve of this diatom is essentially unaffected by addition of bacterial filtrates that are active against other diatoms. By monitoring proteases from the medium using zymography and fluorescence based activity assays we demonstrate that *C. didymus* responds to the presence of the lytic bacteria with the induced production of algal proteases. These proteases exhibit a substantially increased activity compared to the bacterial counterparts. The induction is also triggered by signals in the supernatant of a *K. algicida* culture. Size fractionation shows that only the >30 kD fraction of the bacterial exudates acts as an inducing cue. Implications for a potential induced defense of the diatom *C. didymus* are discussed.

## Introduction

Diatoms (Bacillariophyceae) comprise an abundant group of unicellular microalgae distributed worldwide in marine and freshwater habitats. These algae play a crucial role in the marine ecosystems as central primary producers [1]. Thus, a detailed understanding of the diatom population dynamics is fundamental for a comprehensive view on marine ecology. Factors influencing diatom blooms are diverse, and include abiotic parameters such as temperature or nutrient availability [2] as well as biotic interactions, such as grazing pressure. Such biotic interactions might be mediated by chemical signals as it is found for defense responses to grazers [3], [4] as well as allelopathic interactions with other phytoplankton species [5]–[7]. Additionally bacteria can have a substantial impact on the performance of phytoplankton species including diatoms [8]–[11]. Some bacteria form mutualistic interactions with algae. For example, in the presence of bacteria of the genus *Alteromonas* sp. growth of the toxic dinoflagellate *Alexandrium fundyense* is promoted substantially [12]. Such growth promoting effects of bacteria on different algae can be explained by delivery of vitamins from the bacteria to the algae within a symbiotic interaction [13], [14]. The bacterium *Phaeobacter gallaeciensis* can undergo mutualistic interaction with the coccolithophore *Emiliania huxleyi* by supplying the alga with growth promoting factors and receiving dimethylsulfoniopropionate (DMSP) as a reduced sulfur source. However, when *P. gallaeciensis* recognizes *p*-coumaric acid released by the algae, they alter their metabolism and produce algicides called roseobacticides which kill the algae – a process resulting in a surplus of nutrients for the bacteria [15], [16]. Other bacteria can have detrimental effects on algae by e.g. reducing the swimming motility of dinoflagellates [17] and the growth of diatoms [10], [18]. Enzymes, especially proteases, are frequently the active algicidal factors. The effect of algicides is often species-specific. For instance, *Pseudoalteromonas* sp. release a heat labile compound into the surrounding seawater which inhibits the growth of the dinoflagellate *Alexandrium catenella* but does not affect the growth of the diatom *Skeletonema* sp. or the cyanobacterium *Oscillatoria* sp. [19]. Such inhibitory interactions of bacteria and phytoplankton, mostly dinoflagellates, have often been investigated with the aim of finding a biological control of harmful algal blooms (HABs) [20], [21]. In comparison, the role of inhibitory bacteria on the bloom propagation of non-toxic diatoms has so far been mostly neglected. Diatoms occur in the ocean in complex assemblages [22] with several different species co-existing next to each other. Bacteria might promote this diversity by a species-specific effect of inhibitory substances.

Recently we showed that the release of algicidal proteases by the bacterium *Kordia algicida* is under the control of a quorum sensing mechanism [10]. While several diatom species such as *Skeletonema costatum*, *Thalassiosira weissflogii* and *Phaeodactylum tricornutum* showed a significantly reduced growth after exposure to proteases containing cell-free filtrates of *K. algicida* for approximately 40 h, the cosmopolitan diatom *Chaetoceros didymus* [23], [24] was not inhibited. The reason why *C. didymus* is not susceptible to the enzymes released by *K. algicida* remained elusive. To our knowledge no defense mechanisms explaining the selective mode of action of algicides are currently known, even though cell surface associated polysaccharides were suggested to play a role by protecting cells against proteolytic stress [9].

Here we show that *C. didymus* is indeed resistant to proteolytic attack by *K. algicida* and responds to the presence of the bacteria with induced responses. The resistance may be explained by the fact that *C. didymus* releases additional proteases, induced by excreted proteins of *K. algicida*, which might serve as a chemical defense.

## Materials and Methods

### Algal and Bacteria Culturing

The bacterium *Kordia algicida* strain OT-1 was obtained from the NITE Biological Resource Center (NBRC 100336) and grown at room temperature under constant shaking (80 to 100 rpm) in ZoBell medium (5 g bacto peptone, 1 g yeast extract, 10 mg FePO_4_, 34 g Instant Ocean (Aquarium Systems, Sarrebourg, France) in 1 L ultra pure water). An exponentially growing culture was used to prepare a glycerol stock of this culture which was subsequently used to prepare starting cultures in ZoBell medium before each set of experiments.

*Skeletonema costatum* (RCC 75) was obtained from the Roscoff culture collection, France. *Chaetoceros didymus* was originally isolated by S. Poulet at the Station Biologique in Roscoff, France and maintained in our culture collection. The diatoms were cultivated in artificial seawater [25] buffered at a pH of 7.8 at 15°C under a light/dark rhythm of 14/10 hours and an illumination of 40–45 µmoles photons s^−1^ m^−2^. The final nutrient concentrations in the medium were 620 µM nitrate, 14.5 µM phosphate, and 320 µM silicate.

### Estimating Bacterial and Algal Growth

Bacterial growth was monitored using the optical density at 550 nm (OD) measured with a Specord M42 UV-vis spectrophotometer by Carl Zeiss (Jena, Germany).

The diatom growth was monitored by measuring the chlorophyll a fluorescence with a Mithras LB 940 plate reader (Berthold Technologies, Bad Wildbad, Germany). The fluorescence of 1.5 mL *C. didymus* cultures in 24 well plates (Greiner Bio-One, Frickenhausen, Germany) was measured without any further preparation.

### Cell Free Bacterial Filtrates

Cell free bacterial filtrate were obtained as described [10]. Briefly, exponentially growing *K. algicida* was inoculated into a mixture of artificial seawater and ZoBell medium in a ratio of 10∶1 until the culture reached an OD >0.3. Afterwards, 1 mL of the culture was inoculated into 50 mL of seawater. After 24 h cell free filtrates were obtained by sterile filtration with 0.2 µm polyethersulfone filters (Carl Roth, Karlsruhe, Germany).

### Monitoring Effects of Bacterial Filtrates on Algal Growth

To test for effects of bacterial filtrates on diatom growth 1.125 mL of cell free bacterial filtrate was inoculated with 0.375 mL of exponentially growing *C. didymus* or *S. costatum* cultures in 24 well plates. Controls were run in parallel using seawater instead of bacterial filtrates. The *in vivo* chlorophyll a fluorescence was regularly measured as indicator for algal growth. Five independent biological replicates (i.e. samples from independent cultures) were monitored.

### Measuring Protease Activity

The protease activity of culture filtrates was measured by following the conversion of BIODIPY FL casein (E 6638, Invitrogen, Carlsbad, CA, USA) to fluorescent products [26]. First, 10 µL of *C. didymus* or *S. costatum* culture medium were mixed with 100 µL of digestion buffer (Invitrogen, 200 mM Tris, pH  = 7.8, 2 mM azide) and 100 µL of the dye at a concentration of 10 µg ml^−1^. After incubation for 1 h at room temperature in the dark the fluorescence of the protease products was measured using a Mithras LB plate reader with a 470±5 nm excitation filter and a 510±20 nm emission filter. Four independent biological replicates were monitored.

### Protein Concentration and Size Fractionation for Bioassays

Protein concentrations were analyzed from *K. algicida* and *C. didymus* cultures as well as from cultures of *C. didymus* grown in *K. algicida* conditioned medium. The cultures were sterile filtered using 0.2 µm filters before adding 30 mL of the cell free medium to Amicon centrifugal filter units (Millipore, Billerica, MA, USA) with a molecular weight cut off of 30 kDa as described in the manufacturer’s instructions. The resulting filtrate was concentrated to a final volume of approximately 300 µL representing a 100 fold concentrated protein fraction.

### SDS-polyacrylamide Gel Electrophoresis and Zymograms

The concentrated protein preparations were mixed 1∶1 (vol %) with loading buffer (3 g SDS, 3 mg bromophenol blue, 3 mL glycerol, 3.75 mL 500 mM Tris, pH  = 6.8, volume adjusted to 10 mL with ultra pure water). From each sample, 20 or 30 µL were loaded onto a SDS gel (5% polyacrylamide stacking gel and a 12% polyacryamide separating gel) [27]. The molecular weight of the proteins was estimated based on the comparison to molecular weight standards (Fermentas, St. Leon-Rot, Germany). The gels were run at a voltage of 80 V until the staining reached the separating gel. Afterwards the voltage was increased to 120 V until the loading dye was close to the end of the gel.

After electrophoresis protease activity staining in zymograms was performed according to a modified protocol of Garcia-Carreno [28]. Briefly, the gels were washed in deionized water before incubating in a 0.75% casein Hammerstein (VWR, Dresden, Germany) solution at 4°C for 30 min and at room temperature for 60 min. Afterwards the gels were washed 3 times with deionized water and subsequently incubated in phosphate buffered saline (PBS, pH  = 7.4) for 60 min. After washing the gels again 3 times the proteins were fixed in 12% trichloroacetic acid (VWR) for 30 min. The gels were then stained using Coomassie Brilliant Blue solution for 60 min and destained in methanol/acetic acid/water (415 mL/83 mL/502 mL) for 30 min.

### Effect of Induced *C. didymus* Proteases on *S. costatum*

A bacterial filtrate was obtained as described above. 30 mL of the filtrate were either inoculated with 10 mL fresh seawater or with 10 mL of an exponential growing *C. didymus* culture. After 3 days both treatments were harvested by sterile filtration and supplemented with nutrients to obtain repleted (620 µM nitrate, 14.5 µM phosphate, and 320 µM silicate) culture media. The growth of *S. costatum* was monitored in 24 well plates by inoculating 375 µL exponentially growing *S. costatum* culture to 1.125 mL of the treated filtrates. For a positive control 375 µL of the *S. costatum* culture were inoculated in 1.125 mL seawater.

### Statistical Analysis

The test for statistical significant differences in growth experiments and for protease activity was performed using a two way repeated measures analysis of variance (RM-ANOVA) with Sigmaplot 11 (San Jose, CA, USA). The test of significance was performed using the Tukey post-hoc test implemented in Sigmaplot 11. Differences were accepted as significant for every comparison when P<0.05.

## Results

### *C. didymus* is not Susceptible to *K. algicida* Proteases

**Figure 1:**
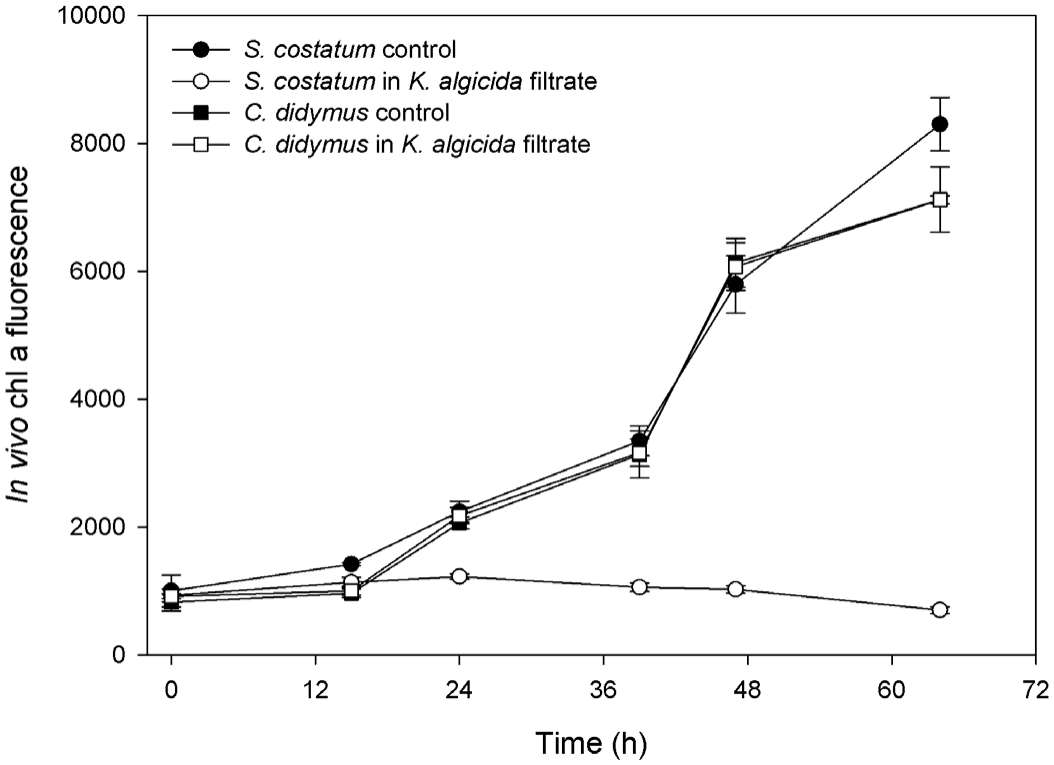
Growth of *C. didymus* and *S. costatum* indicated by in vivo chlorophyll a fluorescence in standard cultures and in cultures supplemented with cell free conditioned *K. algicida* filtrate. Displayed are the mean values+SD (n  = 5).

As recently reported *K. algicida* filtrate was highly algicidal towards *Skeletonema costatum* [10]. A culture of this diatom exhibited directly a drastic reduction of chlorophyll a (chl a) fluorescence compared to the control (P<0.01 after 24 h and onwards, Fig. 1). In strong contrast, the growth of *C. didymus* was not affected by the same filtrate. Over a period of 64 h we could not observe any significant difference in the chl a fluorescence compared to the controls (Fig. 1, P>0.553 at each time point).

### Induced Protease Activity in *C. didymus* Cultures

**Figure 2:**
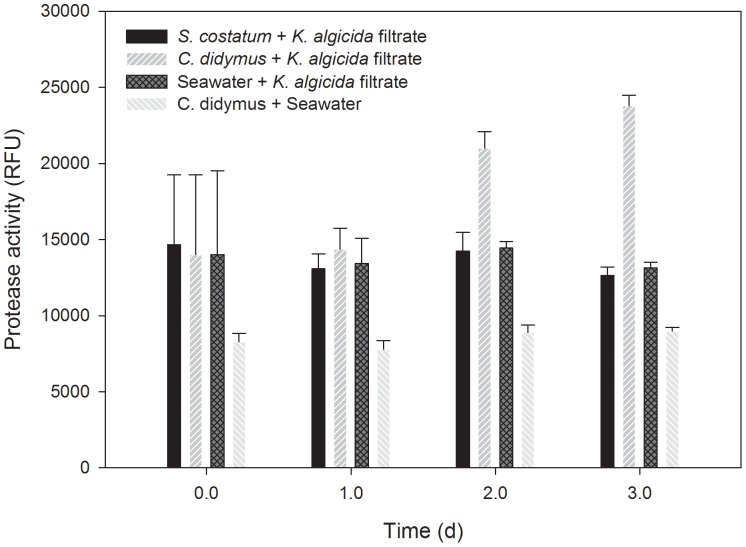
Protease activity in culture medium of the susceptible diatom *S. costatum* and the non-susceptible diatom *C. didymus* after the application of conditioned cell free *K. algicida* filtrate (n = 4). The control shows the protease activity in an untreated *C. didymus* culture.

Because *C. didymus* was not affected by proteases of *K. algicida* we initially hypothesized that it has developed a mechanism to inhibit or degrade the algicidal bacterial enzymes. To test this concept, we measured the protease activity in a *C. didymus* culture over for a period of 3 days after stressing it with cell free bacterial filtrate. We compared this protease activity with an identical treatment of a positive control culture of *S. costatum* that was susceptible to *K. algicida* proteases (Fig. 2). During the first two days of the experiment we did not observe any difference between *S. costatum* and the *C. didymus* cultures (P>0.855). Interestingly, after 2 and 3 days the protease activity was significantly increased in *C. didymus* cultures in comparison to *S. costatum* cultures (P<0.001) indicating a release of additional protease(s) by *C. didymus* and contradicting the initial hypothesis.

### Characterization of Induced Proteases

**Figure 3:**
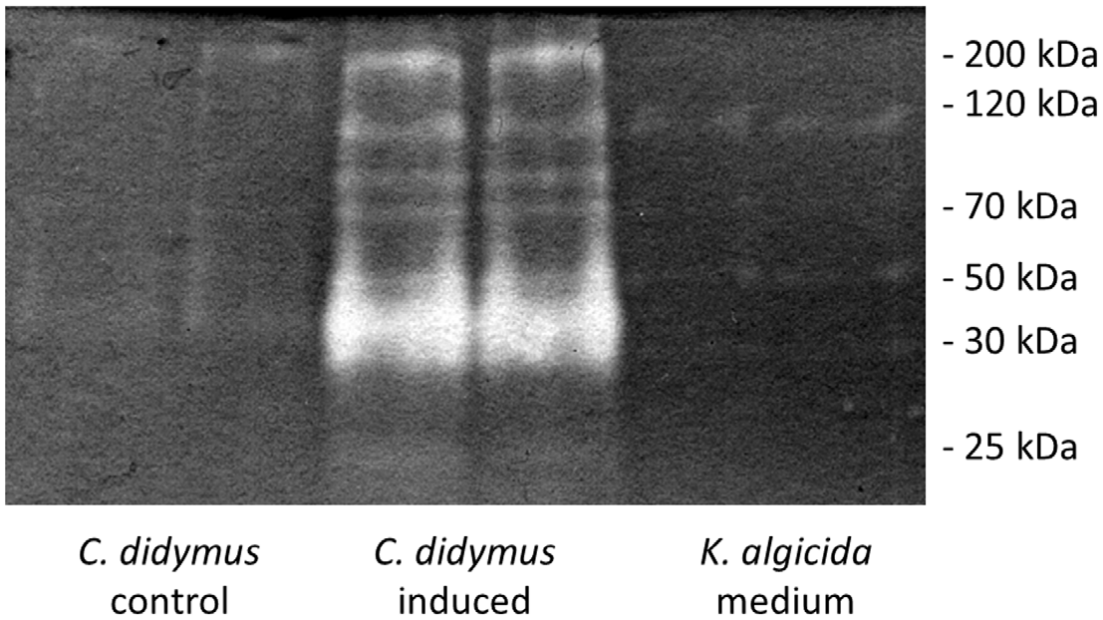
Profiles of released proteases determined by zymography. ”*C. didymus* control” shows protease release of *C. didymus* under standard growth conditions, ”*C. didymus* induced” shows the profile of induced proteases after the application of conditioned cell free *K. algicida* medium, and ”*K. algicida* medium” shows the profile of the *K. algicida* medium used for induction. The respective pairs of lanes show independent biological replicates. The picture has been electronically modified by increasing brightness and contrast.

To characterize protease(s) released by *C. didymus* we performed SDS polyacrylamide gel electrophoresis and stained specifically for proteases in zymograms. A clear zone on a blue background indicates the presence of a protease. Concentrated cell free supernatants of i) *C. didymus* cultures, ii) *C. didymus* cultures conditioned with cell free bacterial filtrate for 3 days and iii) released enzymes from *K. algicida* cultures were investigated. We observed no proteases released by *C. didymus* under standard growth conditions (Fig. 3, lanes 1 and 2). In contrast, when *C. didymus* is grown in cell free filtrates of *K. algicida* we observed several released proteases (Fig. 3, lanes 3 and 4). The molecular weights of these induced proteases were approximately 85, 70 and 35 kDa. The 35 kDa band was most prominent band as indicated by the broad and bright character. In contrast, the extracellular protease profile of *K. algicida* (Fig. 3, lanes 5 and 6) showed only bands with very low intensities at molecular masses of approximately 110 and 50 kDa.

### Initial Characterization of Protease Inducing Signals from *K. algicida*

**Figure 4:**
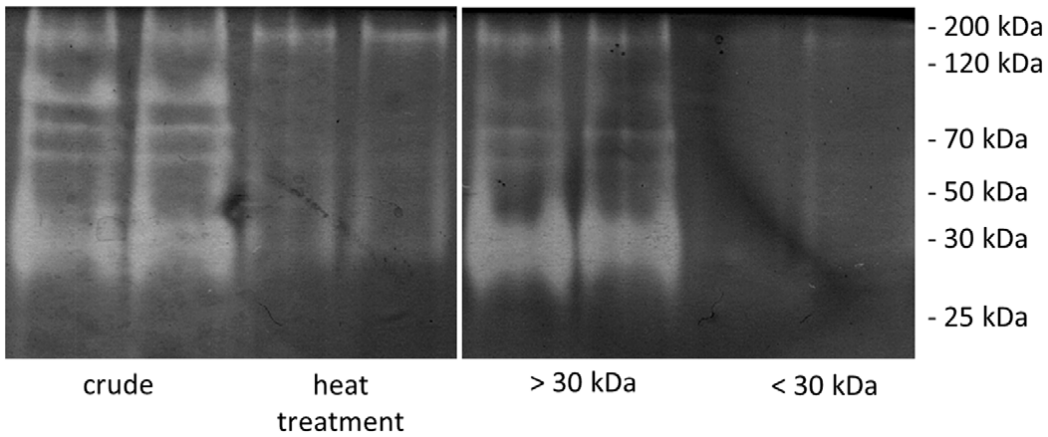
Characterization of the inducing compounds within the bacterial filtrate. ”crude” shows protease profile obtained after the induction of *C. didymus* cultures with unfractionated *K. algicida* filtrate serving as positive control, ”heat treatment” show proteases after the addition of heat treated bacterial filtrate, “>30 kDa” shows the induction after addition of the >30 kDa fraction of the bacterial filtrate, and ”<30 kDa” shows proteases in *C. didymus* medium treated with bacterial filtrates containing metabolites <30 kDa. The picture has been electronically modified by increasing brightness and contrast.

After we verified the protease release from *C. didymus* in response to bacterial cell free filtrates we aimed to characterize the eliciting principles in more detail. Therefore, we compared the extracellular protease profile of *C. didymus* grown in bacterial filtrate (Fig. 4, lanes 1 and 2) with the profile of released proteases of *C. didymus* cultures grown in modified *K. algicida* filtrate. As a modification we used filtrates that were heat treated prior to the addition to *C. didymus* (Fig. 4, lanes 3 and 4), a high molecular weight (>30 kDa, Fig. 4, lanes 5 and 6) and a low molecular weight fraction of the *K. algicida* filtrate (<30 kDa, Fig. 4 lanes 7 and 8). Neither the heat treated filtrate nor the low molecular weight fraction of the filtrate induced the release of proteases (Fig. 4, lanes 3 and 4, and 7 and 8 respectively). In contrast, after application of the high molecular weight fraction of the bacterial filtrate to *C. didymus* we observed a release of proteases with the molecular mass of approximately 85, 70 and 35 kDa (Fig. 4, lanes 5 and 6). This pattern was similar to that observed after induction with unfractionated bacterial filtrate (Fig. 3).

### Potential Inactivation of *K. algicida* Algicidal Activity by *C. didymus*

**Figure 5:**
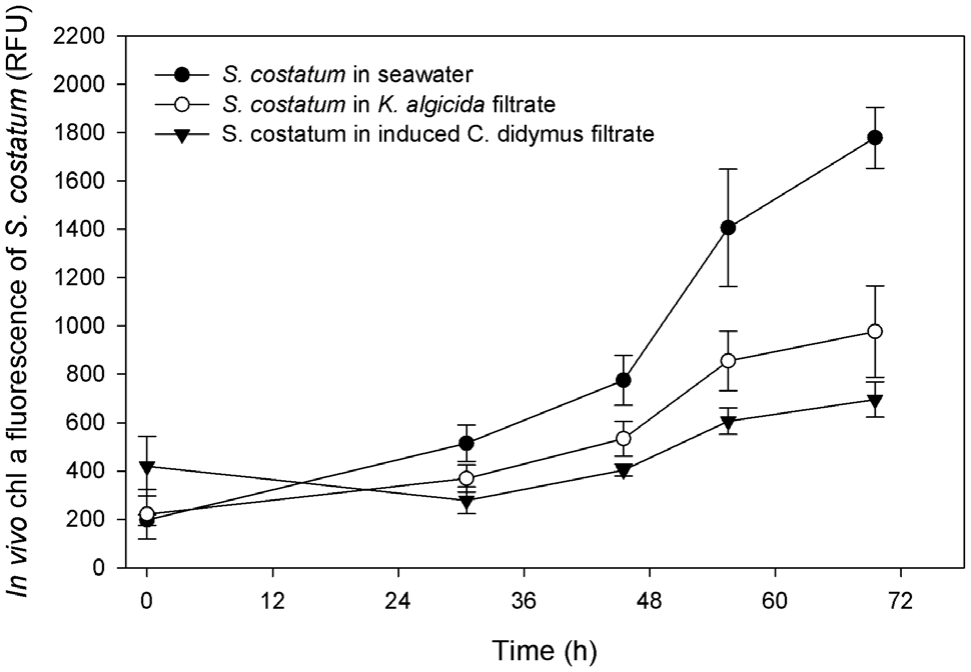
Effect of medium from an induced *C. didymus* culture on *S. costatum*. *In vivo* fluorescence of *S. costatum* grown in the presence of spent *K. algicida* medium (open circles) and medium from an induced *C. didymus* culture (triangles). The control is indicated by black dots (n = 3).

To monitor if *C. didymus* is capable to inactivate the algicidal activity of *K. algicida* medium incubation experiments with *S. costatum* were conducted. As shown above this alga was inhibited in its proliferation by the spent medium from the bacteria culture. If the alga was treated with the same medium that was incubated with *C. didymus* before the start of the experiment algicidal activity was indeed more pronounced (Fig. 5). Since bacteria were not present in this experiment but rather the activity of spent medium was observed, this increased activity points towards an own contribution of *C. didymus* to the overall growth inhibition.

## Discussion

In a previous study the diatoms *Skeletonema costatum*, *Phaeodactylum tricornutum* and *Thalassiosira weissflogii* showed a reduced chl a fluorescence after a two day exposure to *K. algicida* spent medium while *C. didymus* was not affected [10]. To further elucidate the underlying principle of apparent resistance we investigated the effect of *K. algicida* on *C. didymus* in detail. Kinetic experiments revealed that *K. algicida* cell free medium is directly inhibiting the proliferation of *S. costatum* while the growth curves of *C. didymus* in the presence and absence of *K. algicida* medium were virtually superimposable over a three day period (Fig. 1). Initially we hypothesized that *C. didymus* may actively deactivate bacterial proteases for example by protease inhibitors. Therefore we followed the protease activity in the culture medium of both the susceptible diatom *S. costatum* and the non-susceptible diatom *C. didymus* after the application of the *K. algicida* cell free filtrate. We used a fluorescence based method which allows the quantitative evaluation of protease activity covering several types of proteases including serine or metalloproteases [26]. At the beginning of the experiment concentrations of *K. algicida* proteases in *S. costatum* and *C. didymus* cultures were the same and in cultures of *S. costatum* the protease activity remained constant over time (Fig. 2). This can be completely attributed to the stability of the bacterial proteases since measurements of proteolytic activity in seawater incubated with *K. algicida* proteases alone showed similar readings that were also constant over the time of the experiment (data not shown). The protease activity displayed in Figure 2 includes also background fluorescence of the assay which was in the range of 8000 to 10000 RFU. Thus, the relative increase of the induction is higher than the protease activity caused by the bacterial protease.

Interestingly, the *C. didymus* cultures showed a completely different picture falsifying the hypothesis of active protease inhibitors. We observed that indeed the protease activity increased during the experiment suggesting an induced protease release by the alga in response to compounds present in the bacterial filtrates. To further characterize the increased protease activity we profiled the exuded proteases of *C. didymus* using SDS gel electrophoresis combined with zymograpy, a technique that allows the specific detection of proteases in polyacrylamide gels. Adaptation of a previously published method enabled us to detect proteases in a highly sensitive manner sufficient for the monitoring of the culture medium [10]. While *C. didymus* did not excrete any detectable proteases under standard growth conditions, we observed a remarkable induction of several proteases with a molecular weight range of 30 to 200 kDa after the addition of bacterial cell free conditioned medium. This induction can be clearly attributed to a response to bacterial substances because neither *C. didymus* under standard growth condition nor *K. algicida* release a similar protease profile. The protease profiling of *K. algicida* medium revealed only minor staining of proteases compared to induced *C. didymus* medium.

Protease induction observed in *C. didymus* cultures can be attributed to the algae and not to contaminating bacteria. We kept bacterial counts as low as possible by single cell isolation and constant work under sterile conditions, but full axenic conditions could not be reached with healthy *C. didymus* cultures. This indicaties the physiological need of bacteria that might, for example provide essential nutrients [13], [14]. The bacterial cell counts in our study were below a value that could be reliably quantified but single cells were observed occasionally. Under these conditions it might be estimated that bacterial proteins do only contribute to a negligible fraction to the total protein content of the culture. In a comparable study bacterial cell counts in phytoplankton of 10^6^ cells mL^−1^ (a value way above the cell counts in this study) contributed to 0.4% of the total protein content [29]. It is thus very unlikely that detectable amounts of proteins in our zymograms result from bacterial origin.

The influence of bacteria on the exudation of organic matter by diatoms has recently been observed in other studies as well. The diatom *T. weissflogii* requires co-existing attached bacteria to form transparent exopolymer particles (TEP) [30]. Furthermore the release of dissolved organic matter (DOC) in the presence of co-occurring bacteria has been documented for other diatoms [31]. This effect of bacteria on diatom DOC release was highly variable and changed over time with the available nutrient concentration. Interpretation of these experiments is complicated because they were based on the addition of living bacteria that could modify the DOC pool themselves [32]. In a general survey of the growth and release of organic compounds by diatoms in response to bacteria like the induction of extracellular polymeric substances including carbohydrates and proteins was observed in more than half of the 14 tested benthic diatoms. MALDI sequencing of proteins induced in the treatment of the diatom *Phaeodactylum tricornutum* with *E. coli* revealed among others a hit with 60% similarity to a protease from a red alga [33]. These preliminary findings might point towards the fact that an induced protease release in response to bacteria might be more widespread among diatoms.

To characterize the nature of the inducing signal from bacteria we performed a size fractionation of the bacterial filtrate and tested the effect on *C. didymus*. Initially, two fractions (>30 kDa and <30 kDa) generated by centrifugal filter units were tested on their potential to induce protease release in *C. didymus*. Only the high molecular weight fraction showed activity comparable to the crude bacterial extract (Fig. 4), indicating that the release of the diatom proteases is a direct response to biopolymers from the bacteria. Since heat inactivation by boiling reduced the induction significantly it might be concluded that non-denaturated proteins are essential for the induction.

It is a compelling hypothesis that the release of diatom proteases by *C. didymus* is involved in the resistance against *K. algicida*. However due to the identical function of the induced proteases from *C. didymus* and the algicidal proteases from *K. algicida* this hypothesis is hard to verify. If the susceptible diatom *S. costatum* was tested with the filtrate of the bacteria cultures directly or after incubation with *C. didymus* an even higher algicidal activity was observed in the diatom treated medium (Fig. 5). Even if bacterial exudates were inactivated after treatment with *C. didymus* the up-regulated protease from the diatoms themselves might have an (additional) adverse effect on *S. costatum*. The ultimate prove if the observed resistance is due to the induced protease up-regulation is thus still open.
